# Complications Following Irinotecan-Loaded Microsphere Chemoembolization of Colorectal Metastatic Liver Lesions Associated with Hepatic-Artery Branch Temporary Stasis

**DOI:** 10.3390/curroncol28030211

**Published:** 2021-06-20

**Authors:** Marcin Szemitko, Elzbieta Golubinska-Szemitko, Jerzy Sienko, Aleksander Falkowski

**Affiliations:** 1 Department of Interventional Radiology, Pomeranian Medical University, 70-111 Szczecin, Poland; bakhis@hot.pl; 2 Department of General and Dental Diagnostic Imaging, Pomeranian Medical University, 70-111 Szczecin, Poland; e.golubinska@gmail.com; 3 Department of General and Transplant Surgery, Pomeranian Medical University, 70-111 Szczecin, Poland; jsien@poczta.onet.pl

**Keywords:** DEB, chemoembolization, near stasis, post-embolization syndrome, SN-38

## Abstract

Chemoembolization with irinotecan-loaded microspheres has proven effective in the treatment of unresectable liver metastases in the course of colorectal cancer (CRC). Most researchers recommend slowly administering the embolizate at the level of the lobar arteries, without obtaining visible stasis. However, there are reports of a relationship between postoperative embolizate retention in metastatic lesions and the response to treatment. To retain residual embolizate throughout the entire neoplastic lesion requires a temporary flow stop (stasis) within all supply vessels, which may cause temporary stasis in subsegmental or even segmental vessels. Objective: To assess the risk of complications and post-embolization syndrome severity following chemoembolization of CRC metastatic liver lesions with microspheres loaded with Irinotecan, with regard to hepatic-artery branch level of temporary stasis. Patients and methods: The study included 52 patients (29 female, 23 male) with liver metastases from CRC, who underwent 202 chemoembolization treatments (mean: 3.88 per patient) with microspheres loaded with 100 mg irinotecan. Postembolization syndrome (PES) severity and complication occurrence were assessed with regard to the hepatic-artery branch level of temporary stasis. Adverse events were assessed according to Cancer Therapy Evaluation Program Common Terminology Criteria for Adverse Events. Results: Median survival from the start of chemoembolization was 13 months. From 202 chemoembolization sessions, 15 (7.4%) significant complications were found. The study found a significant relationship between the branch level of temporary stasis and the presence of complications (*p* < 0.001), with the highest number of complications observed with temporary stasis in segmental vessels. PES was diagnosed after 103 (51%) chemoembolization treatments. A significant association was found between PES severity and the branch level of temporary stasis (*p* < 0.001). Conclusions: The branch level of temporary stasis affected the severity of post-embolization syndrome. A significant association was found between the branch level of temporary stasis obtained in chemoembolization procedures and the presence of complications. The apparent lack of change in numbers of complications when stasis was applied at tumor supply vessels or subsegmental arteries may indicate the safe use of temporary stasis in some cases where colorectal cancer metastases are treated. Further research is needed to determine the most effective chemoembolization technique.

## 1. Introduction

Colorectal cancer (CRC) is one of the most common causes of cancer death. Approximately 50–60% of patients diagnosed with CRC develop colorectal hepatic metastases (CRHM) [1]. Among possible therapeutic methods, surgical treatment of metastatic lesions offers the longest survival time but is possible in only around 10–15% of patients [2,3]. Moreover, in ~50% of patients after liver metastases resection, there is recurrence of metastatic lesions. In patients who are not eligible for surgery, options exist for standard chemotherapy or chemoembolization, the latter often giving greater efficacy [4,5].

Due to the fact that CRC liver metastases are almost exclusively supplied by branches of the hepatic artery, the embolization of these with irinotecan-loaded microspheres results in delivery of a high dose of the chemotherapeutic agent directly to the lesions, giving increased exposure to irinotecan and, at the same time, lower systemic exposure. Unlike embolization in the case of hepatocellular carcinoma (HCC), the vast majority of researchers recommend that, for CRHM, the embolizate be administered slowly at the level of the lobar branches of the hepatic artery. The aim has been to deliver the irinotecan microspheres to the entire liver parenchyma without an embolic effect of stasis in the hepatic artery branches, the presence of which might increase the frequency of side effects. However, there are reports that suggest that retention of the mixture of contrast and irinotecan-loaded microspheres in tumors affects response to treatment. It has been shown that the active metabolite of Irinotecan, SN-38, is more efficiently converted and released if the flow in the tumor vessels is stopped [6]. Moreover, positron emission tomography/computed tomography (PET-CT) studies have shown a significant association between the area of embolizate retention in CRC metastases and the response to treatment [7]. Temporary stasis produced during Drug-Eluting Bead Transarterial Chemoembolization (DEB-TACE) procedures of liver vessels, by preventing rapid leaking of irinotecan and its metabolite SN-38 from the tumor, may affect treatment efficacy [8].

**Figure 1:**
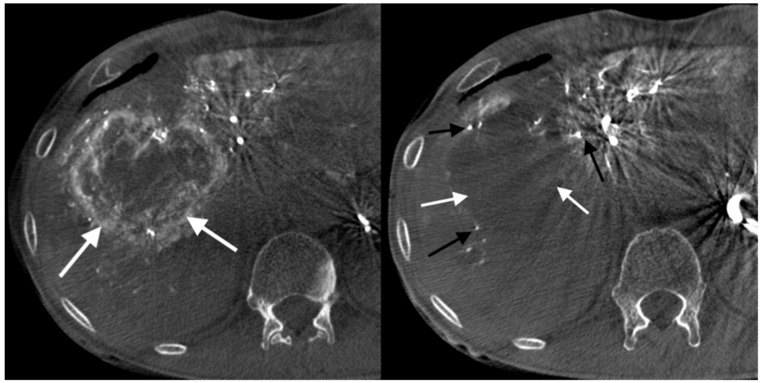
Cone-beam computed tomography image was performed through the SIM catheter in the coeliac artery using 45 mL Iodixanolum (320 mg I/mL) with flow rate of 5 mL/s. Devascularization of a colorectal cancer metastatic liver focal lesion. Left, before the procedure: visible pathological tumor vessels (white arrows). Right, three weeks after the procedure: preserved patency of subsegmental branches (black arrows) and almost complete devascularization of tumor vessels (white arrows).

In order to retain the embolizate over the largest possible area of the neoplastic lesion, it is necessary to stop (to create stasis) or slow the blood flow in as many blood supply vessels as possible, at least temporarily. Most authors propose a temporary slowdown in blood flow (to give so-called “near stasis”), with return to normal flow within 2 to 5 heartbeats. However, taking into account the multiple and complex vascular supplies of metastatic lesions, lobar or segmental administration (preferred by most researchers) of the embolizate may lead to consequent formation of temporary stasis within the tumor supply vessels or even subsegmental or segmental arteries. This stasis reflects a temporary inhibition of blood flow, most commonly visible during surgery. However, arteriography performed during subsequent DEB-TACE procedures frequently shows that segmental and subsegmental branches, initially affected by the temporary stasis, have preserved patency (Figure 1).

Although some authors have treated stasis as an end point of chemoembolization [9] or as an unintentional side-effect from the procedure [10], there are no studies in the literature assessing the dependence of side effects, and tolerance of DEB-TACE treatments with irinotecan, on the branch level of temporary stasis in the liver vessels.

### Objectives

The objective of this study is to assess the risk of complications and the severity of post-embolization syndrome following chemoembolization of colorectal cancer metastatic liver lesions, with regard to branch level of temporary embolizate stasis in hepatic arteries.

## 2. Materials and Methods

This retrospective study assessed the chemoembolization of liver metastatic lesions in the course of CRC surgery with procedures performed between November 2016 and March 2019. The study was approved by the Bioethics Committee of the Pomeranian Medical University, Szczecin, Poland. The study analyzed 52 patients (29 women and 23 men) who underwent a total of 202 chemoembolization treatments using microspheres loaded with a cytostatic topoisomerase I inhibitor: 100 mg of Irinotecan per treatment.

Qualification for the procedure was performed according to the recommendations of the European Society of Medical Oncology (ESMO) after consultation with a specialist in oncology, on the basis of CT and/or magnetic resonance imaging (MRI) of the abdominal cavity and laboratory results.

Indications for treatment were: unresectable and dominant liver metastases with progression after prior systemic chemotherapy, Eastern Cooperative Oncology Group (ECOG) performance level <2, no evidence of liver failure, and age over 18 years. Exclusion criteria were: ECOG scale >2, liver failure, ascites, bilirubin levels above 3 mg/dL, involvement of more than 50% of the liver parenchyma, renal failure (creatinine above 2 mg/dL), thrombocytopenia below 50,000/mcL, and allergy to contrast.

The treatment was performed according to a schema that included four procedures (or two in the case of only one lobe of the liver), at intervals of 2–3 weeks, with alternating embolization of the branches of the right or left hepatic artery and additional arteries supplying the liver lesions (the first side chosen at random). Embozene Tandem 100 µm microspheres (CeloNova Biosciences, now Varian Medical Systems, Palo Alto, CA, USA) were used. After Irinotecan was loaded onto the microspheres, the supernatant was removed, and the remaining loaded microspheres were mixed with 10 mL of contrast agent (Iodixanolum 320 mg I/mL). The procedures were performed by interventional radiologists with skill certificates and at least six years of experience in interventional radiology.

On the day of the procedure, each patient received prophylactic antibiotics, steroids, proton pump inhibitors, and, additionally, an antiemetic and infusion of 1000 mL of 0.9% NaCl as ordered by the participating anesthesiologist according to hospital guidelines.

### 2.1. Procedure

The right or left common femoral arteries were accessed using the Seldinger puncture method. Then the celiac trunk was catheterized (in the case of an anatomical variant of the hepatic artery visible from a previous CT examination, other visceral arteries were also catheterized) using a SIM 5F catheter (Cordis, Santa Clara, CA, USA) and arteriography and cone-beam CT (Dyna-CT) examination were performed.

Hepatic vascularization and metastatic lesions were then assessed, and depending on their location and the appearance of the supply vessels, the hepatic artery branches (distal to the exit of the cystic artery) were selectively catheterized using a Progreat^®^ 2.7F microcatheter (Terumo, Tokyo, Japan). Prior to each administration of microspheres, selective arteriography was performed to verify the position of the microcatheter and exclude any deviance. Each administration of microspheres was preceded by a catheter injection of 2–5 mL of lidocaine. Then, under fluoroscopy, the mixture of microspheres and contrast medium was slowly administered (at a rate of about 1 mL/min), while taking care that there was no reflux proximal to the catheter tip. If the lesions were numerous and had multiple vascularization, administration of the embolizate at the level of the lobar or segmental artery was preferred.

Once slow flow (giving “near-stasis”) was achieved in the tumor supply vessels, the administration of microspheres was stopped. In the case of large lesions, i.e., identified by arteriography to cover the vast majority of the area vascularized by a given subsegmental or segmental branch, the tumor supply vessels were initially embolized in order to deliver a larger dose of the drug to this area. In this case, administration of the embolizate was carried out until “near-stasis” was achieved at the level of the selectively catheterized subsegmental or, less frequently, segmental branch.

After waiting for one minute, the level of stasis in the embolized vessels was assessed and recorded using fluoroscopy. The waiting time of one minute after the end of embolizate administration allowed, on the one hand, stabilization of the changing temporary stasis in the hepatic artery branches but, on the other hand, did not significantly prolong the procedure, which could affect the risk of microcatheter occlusion and/or the patient’s tolerance to the procedure.

The position of the microcatheter was then changed more proximally in order to embolize remaining regions of the liver. Most procedures were completed following administration of the embolizate with the microcatheter tip in the lobar artery, in order to apply the embolizate to the entire liver area to be treated. Only eight treatments were completed at the level of the segmental arteries of the right lobe due to deviation of a cystic artery.

**Figure 2:**
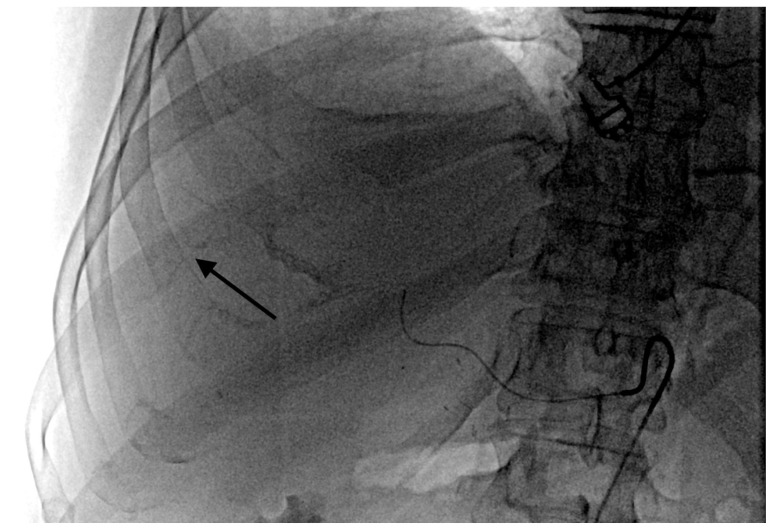
Temporary stasis at the level of the tumor supply vessels (arrow).

**Figure 3:**
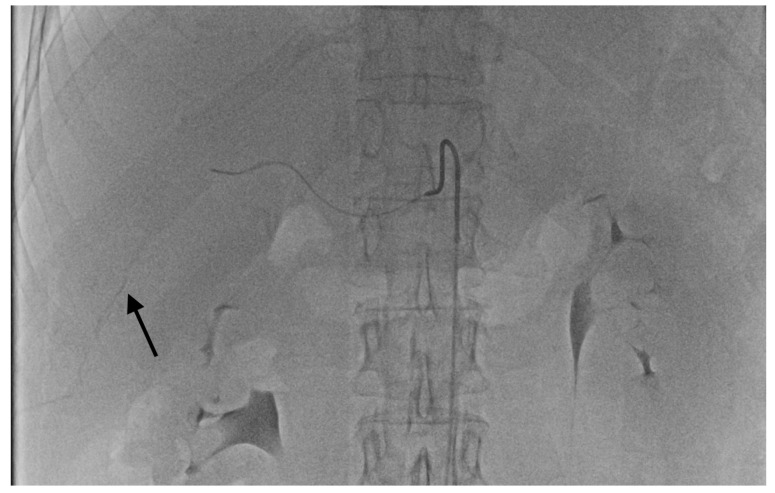
Temporary stasis at the level of subsegmental vessels of right hepatic artery (arrow).

**Figure 4:**
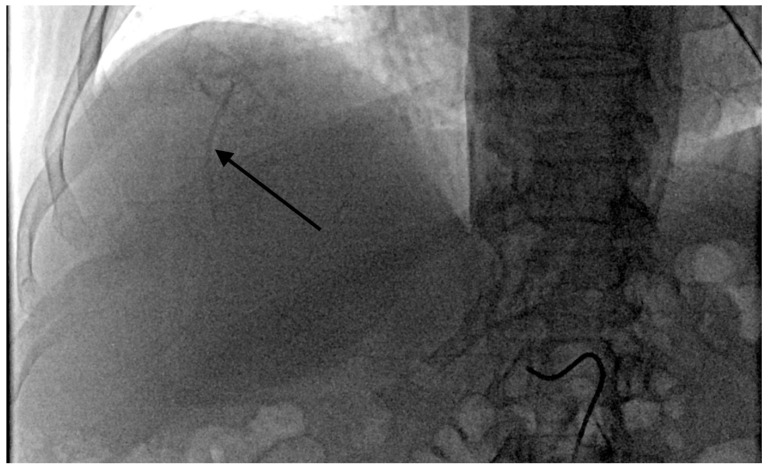
Temporary stasis at the level of segmental vessels of right hepatic artery (arrow).

The grade of deposition of the contrast and microsphere mixture at the vessel level after one minute was used to define the degree of temporary stasis:

Grade 1: no stasis. No visible embolizate retention (or only in areas visible at tumor supply vessel level).
Grade 2 stasis. Embolizate contrast at the level of tumor supply vessels (Figure 2).
Grade 3 stasis. Embolizate contrast in subsegmental vessels (Figure 3).
Grade 4 stasis. Embolizate contrast in segmental vessels (Figure 4).

In the case of varying degrees of stasis in several arteries, the highest degree of stasis was taken into account for the analysis.

Assessment of the degree of stasis achieved was performed on the basis of recorded images, independently by three interventional radiologists, each of whom had at least six years of experience in interventional radiology.

During the procedure, the patient was under the care of an anesthesiologist. Pain, during and after the procedure, was controlled with continuous opioid infusion (20 mg morphine daily) plus non-steroidal anti-inflammatory agents. Prophylactic twice-daily antiemetics (ondansetron 8 mg I.V.), dexamethasone 8 mg I.V., and an antibiotic (cefazolin 1 g I.V.) were administered twice a day. Most patients were discharged from the hospital the day after surgery.

### 2.2. Adverse Event Assessment

Complications and the post-emebolization syndrome (PES) were assessed via observation of the patient during hospitalization and follow-up examinations after 7 and 14 or 21 days after the procedure. Adverse events and complications occurring periprocedurally and within 30 days after surgery were assessed using the standards and terminology of the Cancer Therapy Evaluation Program Common Terminology Criteria for Adverse Events, Version 5.0.

Post-embolization syndrome was assessed according to the following scale:

Pain was assessed according to a 0–10-point scale, with 0 as no pain and 10 as very severe pain.

0: no symptoms of post-embolization syndrome.
1: moderately severe post-embolization syndrome, not requiring additional treatment: moderate pain (1–5 points; pain scale given above), and increased body temperature up to 38 °C.
2: severe post-embolization syndrome requiring additional treatment: severe pain (6–10 points), fever over 38 °C, nausea, and vomiting.

### 2.3. Statistical Analyses

Descriptive statistics of the studied variables were given as arithmetic means and standard deviations or as medians and range. The relationships between stasis and the presence or absence of complications or the severity of PES were assessed using Pearson’s Chi-squared tests. A *p*-value of <0.05 was considered significant. Overall survival (OS) was calculated using the Kaplan–Meyer method from the date of the patient’s first DEB-TACE treatment to either the date of the last follow-up visit for that patient or the patient’s death. All statistical analyses were performed using a commercial program (Statistica, ver. 13.1; StatSoft Polska, Krakow, Poland).

## 3. Results

### 3.1. Patient Characteristics

All patients enrolled in the study (*n* = 52) had unresectable CRC metastases in the liver, and six patients also had lung metastases with the liver being the dominant site of metastasis. Most (41) patients had metastases in both lobes of the liver (mean: 5.4 metastases per lobe) and 11 had metastases limited to one liver lobe (mean: 1.9 metastases per lobe). In 47 patients, the degree of involvement of the liver parenchyma was below 25%, and only in five patients was it within the range of 25–50%. Each patient had been previously treated with at least one systemic chemotherapy regimen and 41 patients had received at least two lines of chemotherapy with evidence of progression (Table 1).

### 3.2. Chemoembolization

A total of 202 chemoembolization procedures were performed in 52 patients. In 11 patients with one lobe involvement, 22 chemoembolization procedures were performed. The remaining 41 patients (with two lobes affected) underwent 180 chemoembolization treatments. The technical success was 100% (Table 2).

**Figure 5:**
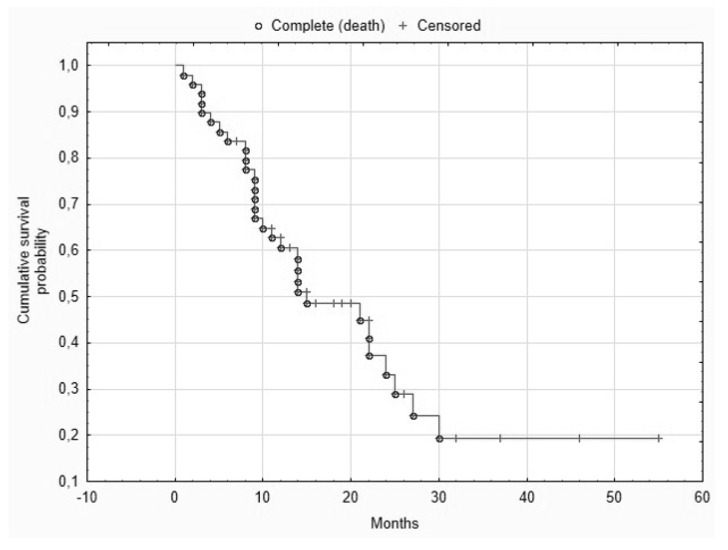
Kaplan–Meier survival analysis for all patients.

The median survival time after chemoembolization was 13.2 months. One-year survival was 63% and two-year survival was 33% (Figure 5).

Post-embolization syndrome was diagnosed following 103 (51%) DEB-TACE treatments. In 67 treatments, the severity of PES was low, while after 30 treatments, strong PES symptoms appeared requiring additional treatment (Table 3).

### 3.3. Adverse Events

From the 202 chemoembolizations performed, significant complications were found in 15 (7.4%). Follow-up imaging studies showed signs of dilatation of the bile ducts in four patients, also with occlusion of the segmental branch of the hepatic artery. In all of these patients, the branch level of temporary stasis was at the segmental artery level. This shows that proximal stasis may in some cases be unresolved and cause damage to the biliary plexuses and even occlusion of the segmental branch of the hepatic artery.

In two cases, an anaphylactic reaction occurred with moderate hypotension, reddening of the skin, and coughing during the procedure, which resolved after intervention of the anesthetic team. One patient experienced a septic episode with liver abscess two weeks after the last treatment, which was treated successfully with antibiotic therapy without drainage. Three patients with temporary stasis at the segmental level showed signs of cholecystitis on ultrasound, which resolved after conservative treatment. Two patients had features of hepatic decompensation with the appearance of ascites. Three patients had leukopenia (<2000/mm^3^) 14 days after surgery, requiring an additional week for the next TACE session.

There were no deaths in the periprocedural period or within 30 days of the procedure.

A statistically significant relationship was found between the branch level of temporary stasis and the presence of complications (chi-squared value = 30.3, df = 3, *p* < 0.001). The highest number of complications was recorded in cases of temporary stasis at the level of segmental vessels (Table 5). However, no association was found between the number of complications for temporary stasis when restricted to the levels of the tumor, tumor supply vessels, and subsegmental arteries (chi-squared value = 0.329, df = 2, *p* < 0.001). (Table 5).

## 4. Discussion

Chemoembolization, with microspheres loaded with irinotecan (Drug-Eluting Beads loaded with Irinotecan), is a relatively well-tolerated palliative therapy in the treatment of liver metastatic lesions from the course of CRC, with a 30-day mortality of about 1.2% [11,12] and a risk of serious complications of around 1.6–7.2% [13,14].

The most common side effect of a drug-eluting bead chemoembolization procedure is post-embolization syndrome, the frequency of which is estimated at 15–90% [15,16].

The most common significant complications of chemoembolization include cholecystitis and liver failure. Less frequent complications include segmental biliary dilatation, leukopenia, thrombocytopenia, liver abscesses, hepatic-artery-branch thrombosis, and vascular complications or complications related to the migration of embolization material beyond the blood vessels of the liver. Migration to the gastroduodenal artery carries a risk of pancreatitis as well as gastro-duodenitis, inflammation, and bleeding [17].

The risk factors for side effects include: the number of DEB-TACE procedures, the administered dose of irinotecan over 100 mg per procedure, the omission of a preceding intra-arterial injection of lidocaine, and the presence of reflux or stasis in the embolized vessels [18]. It is recommended that the embolizate should be administered slowly, avoiding reflux and permanent stasis in the vessels, which can damage the biliary plexuses and dilate the bile ducts.

There is also an association between hypoxia caused by the arrest of blood flow in tumor vessels and increase in the serum concentration of vascular endothelial growth factor (VEGF), which may contribute to the growth of neoplastic lesions [19,20,21]. On the other hand, association has been found between the area of embolizate deposition in tumors with subsequent hypometabolic areas visible by PET-CT. Studies have shown a stronger conversion of irinotecan to its metabolite SN-38 (7-Ethyl-10-hydroxy-camptothecin), in hepatocytes with a higher activity of carboxylesterase, presumably due to the generated hypoxia and a decrease in pH within tumor cells [22,23].

Most SN-38 is formed in the liver parenchyma from where it diffuses into surrounding tumor cells [24]. In large tumor foci, such diffusion is difficult, and the activity of carboxylesterase in tumor cells is lower than in healthy liver parenchyma. In addition, extensive arterial vascularization of the tumor contributes to a greater washout of Irinotecan and/or SN-38. This may lead to shorter and less exposure of the neoplastic cells to SN-38 and a poorer therapeutic effect. Temporary blockage of the tumor vascular flow might increase the tumor’s exposure to SN-38 and explain the strong reduction seen in PET-CT-visualized metabolism in areas that have retained embolizate. To obtain the largest possible retention area of the embolizate in the case of a large and extensive neoplastic lesion requires a temporary stop of blood flow in as many tumor supply vessels as possible. Due to the multiple vessels and complex vascularization of such lesions, direct cannulation of all the vessels is not possible and requires a more proximal, but still superselective, administration of the embolizate at the level of the subsegmental or sometimes segmental arteries. In an area of the liver occupied by neoplastic infiltration the biliary plexuses have mostly degenerated, so even superselective chemoembolization does not have much effect on these.

It has not been determined so far whether an increase in vascular endothelial growth factor (VEGF) is mainly caused by tumor hypoxia or whether hypoxia of the biliary plexuses is of greater importance, as these are exposed to partial ischemia in each chemoembolization procedure [25].

In our study we have assessed, using chemoembolization with irinotecan-loaded microspheres, the relationship between the hepatic-artery branch level of temporary stasis with the occurrence and severity of side effects. We have shown a significant association between the branch level of temporary stasis and the severity of post-embolization syndrome after the chemoembolization procedure, which is important for the tolerance of the procedure by patients. However, the observed complication rate was small and consistent with conclusions from large multicenter studies regarding the safety of chemoembolization treatments x. We have found a statistically significant association between the numbers of complications with the branch level of temporary embolizate stasis, which is consistent with the opinion of the majority of researchers.

The number of complications was significantly higher but only in the case of stasis at the level of segmental vessels. In our opinion, it is possible to safely use temporary stasis at the level of tumor supply and subsegmental vessels in order to obtain a better therapeutic effect in strictly defined cases, i.e., with more extensive metastatic lesions. However, further research is required to confirm the beneficial therapeutic effects of temporary stasis at the various branch levels.

## 5. Conclusions

The branch level of temporary stasis affected the severity of post-embolization syndrome. A significant association was also found between the branch level of temporary stasis obtained in chemoembolization procedures and the presence of complications. The apparent lack of change in numbers of complications when stasis was applied at tumor supply vessels or subsegmental arteries, with similar numbers to that with no stasis or tumor retention only, may indicate the safe use of temporary stasis in some cases where colorectal cancer metastases are treated. Further research is needed to determine the most effective Irinotecan-eluted bead chemoembolization technique.

### Limitations

1. The study was not a randomized trial but a retrospective analysis. 2. The study would have greater validity if the number of patients in the study group was greater. 3. In the analysis of mortality, all causes of death were taken into account, which did not completely distinguish actual cancer progression from other causes of death.

## Figures and Tables

**Table 1:** Patient characteristics.

Parameter	Value
Number of patients	52
Age, median (range)	67.3 (32–83)
Gender, male/female (*n*)	23/29
ECOG status: 0/1 (*n*)	38/14
Colon/rectal cancer (*n*)	40/12
Bilobar/unilobar metastases (*n*)	41/11
Number of liver metastases, median (range)	5.4 (1–14)
Extent of liver involvement (*n*, <25%/>25%)	47/5
Extrahepatic metastasis (*n*, %)	6 (11.5%)
**Number of lines of prior systemic chemotherapy:**	
**(number of patients)**	
None	0
1	11
2	30
3	11
Prior liver surgery/ablation (*n*)	7/0
Prior locoregional therapy (*n*)	0

**Table 2:** Technical details of therapy with drug-eluting microspheres (100 μm) loaded with irinotecan.

Parameter	Value
Total number of treatments (*n*)	202
Number of treatments per patient: mean (range)	3.88 (1–8)
**Treatment location (*n*):**	
Right	94
Left	108
**Branch level selected (*n*):**	
Lobar	29
Segmental	75
Subsegmental	91
**Branch level with temporary stasis (*n*):**	
No stasis/tumor retention only	36
Tumor supply vessel	65
Subsegmental branch(es)	79
Segmental branch(es)	22

**Table 3:** Branch level of temporary stasis associated with incidence and severity of postembolization syndrome (PES).

Branch Level of Stasis	Total Number of Treatments (*n*)	PES Symptoms (Number of Treatments)		
		None	Mild	Severe
No stasis or tumor retention only	36	32	2	2
Tumor supply vessel	65	39	24	2
Sub-segmental artery	79	25	39	15
Segmental artery	22	3	8	11

A significant association was found between the branch level of stasis and the severity of PES (chi-squared value = 55.1, df = 6, *p* < 0.001), the intensity of which increased with the increase in the stasis branch level (Table 4).

**Table 4:** Branch level of temporary stasis associated with mean severity of postembolization syndrome (PES).

Branch Level of Stasis	PES Mean Severity *
No stasis or tumor retention only	1.17
Tumor supply artery	1.26
Sub-segmental	1.55
Segmental	1.72
Total PES mean	1.425

* Compared using Pearson’s chi-squared test; Significance: *p* < 0.05; chi-squared = 55.1, df = 6, *p* < 0.001.

**Table 5:** Branch level of temporary stasis with the incidence of adverse events.

Number and Grades of Adverse Events			
Branch level of stasis	Adverse events grade		Total
	G 2	G 3	
No stasis/tumor retention only	0	1	1
Tumor supply vessel	2	1	3
Subsegmental artery	2	1	3
Segmental artery	4	4	8

## Data Availability

The data presented in this study are available on request from the corresponding author.
